# An Irreversible Radiological Finding of Lentiform Fork Sign in a Patient With Uremic Encephalopathy: A Case Report

**DOI:** 10.7759/cureus.44850

**Published:** 2023-09-07

**Authors:** Ahmed Ali, Saeed Arif, Maroosha Khan, Laiba Khan

**Affiliations:** 1 Internal Medicine, Fauji Foundation Hospital, Rawalpindi, PAK; 2 Neurology, Fauji Foundation Hospital, Rawalpindi, PAK

**Keywords:** metabolic leucoencephalitis, bilateral basal ganglia hyperintensities, altered mental status, uremic encephalopathy, lentiform fork sign

## Abstract

Basal ganglia are highly metabolically active deep gray matter structures that are commonly affected by toxins, metabolic abnormalities, and systemic, degenerative, and vascular conditions. Basal ganglion affected by uremic encephalopathy can typically result in a “Lentiform fork sign” on T2-weighted imaging (T2WI) and a fluid-attenuated inversion recovery (FLAIR) sequence of magnetic resonance imaging (MRI). This sign represents bilateral symmetrical hyperintensities in the basal ganglia surrounded by a characteristic hyperintense rim demarcating the lentiform nucleus from surrounding structures. This finding is also reported in other conditions resulting in metabolic acidosis from any cause, e.g., diabetic ketoacidosis, organic acidemias, dialysis disequilibrium syndrome, and drugs like metformin. In an appropriate clinicopathological context, the presence of this sign helps in the accurate diagnosis of uremic encephalopathy. The peculiarity lies in the reversible nature of these lesions and their affective response to treatment. However, sometimes these lesions may not be reversible. We present the case of a 60-year-old female who presented to the ER with chief complaints of fever and altered sensorium. Laboratory workup revealed deranged renal function tests (RFTs) and leukocytosis with pyuria. MRI of the brain showed bilateral basal ganglion hyperintensities on T2WI/FLAIR images characteristic of the lentiform fork sign. Therefore, a diagnosis of uremic encephalopathy due to acute kidney injury (AKI) secondary to septic shock from urosepsis was made. The patient was managed conservatively with IV hydration and antibiotics. Remarkable clinical recovery was seen over three weeks. The patient was stable on a five-month follow-up. However, the repeat MRI did not show resolution of the lesions.

## Introduction

Uremic encephalopathy (UE) is a metabolic abnormality commonly encountered in patients with acute kidney injury (AKI) and end-stage renal disease (ESRD). Basal ganglia (BG) are characteristically involved in UE [1]. Encephalopathy in patients with renal failure is commonly attributed to factors such as uremia, sepsis, hypertension, structural abnormalities, and disruptions in electrolyte levels. Its presentation typically includes symptoms such as mild confusion, abnormal movements, tremors, hand flapping, and, in extreme cases, convulsions and coma [2]. It is postulated that the uremic toxins' cytotoxic and vasogenic effects disrupt the blood-brain barrier. Uremic toxins, like guanidine compounds, might also be responsible for an imbalance between excitatory and inhibitory neurotransmitters. On top of this, destructive and demyelinating lesions have also been proposed to be responsible for the pathogenesis [1]. The magnetic resonance imaging (MRI) findings show a wide range of specific features, mainly involving three main areas of the brain: (1) BG, (2) cortical and subcortical areas, and (3) multiple white matter areas [3]. Recently, the presence of lentiform fork sign (LFS) on MRI has been increasingly recognized as a meaningful imaging sign for an accurate diagnosis of UE. The hyperintense rim delineating the putamen on the T2-weighted imaging (T2WI) sequence of MRI in LFS reflects the edema of these regions [3]. Underlying metabolic acidosis is suspected to be a common trigger behind these lesions, as it has been observed in patients with normal renal function with metabolic acidosis secondary to other causes [4]. The involvement of BG in UE is more commonly seen in patients with diabetes mellitus [2,3]. The clinical symptoms and radiological lesions usually show complete or partial resolution by regulating dialysis sessions, treating underlying metabolic acidosis and sepsis, controlling blood glucose levels, and removing harmful drugs like metformin [2].

## Case presentation

A 60-year-old female patient with hypertension and a past medical history of stroke (right middle cerebral artery (MCA) lacunar infarct with full neurological recovery) and depressive illness disorder presented to the hospital with fever, fluctuating consciousness, and altered sensorium for the past five days. The examination showed an axillary temperature of 100.0°F, and the blood pressure was raised to 160/80 mmHg, with a heart rate of 124 beats per minute and a SpO_2_ of 94% on room air. Glasgow Coma Scale (GCS) score was 5/15, and pupils were bilaterally reactive. The patient exhibited hypertonicity, exaggerated reflexes, and bilateral extensor plantar responses; however, the cranial nerve examination was standard. There was no neck rigidity, and Kernig's and Brudzinski's signs were negative. Chest auscultation was significant for bilateral coarse crepitus. A fundoscopy was not performed. The rest of the systemic examination was insignificant. A complete blood count showed leukocytosis with a white blood cell count of 23 x 10^9^/L. Renal function tests (RFTs) were deranged with creatinine of 544 µmol/L and urea of 20.4 mmol/L with pyuria (urine routine/examination (R/E) showed 18-20 pus cells per high power field (HPF)) (Table 1).

**Table 1:** Patient's baseline labs at the presentation. Hb: hemoglobin, PLT: platelets, Na: sodium, Cl: chloride, K: potassium, ALT: alanine transaminase, AST: aspartate transaminase; HCO_3_: bicarbonate.

Labs	Results
Hb	11.7 g/dL
PLT	332 x 10^9^/L
Na^+^	139 mmol/L
Cl^-^	101 mmol/L
K^+^	4 mmol/L
Bilirubin	16 µmol/L
ALT	41 units/L
ALP	185 units/L
pH	7.29
HCO_3_	18 mEq/L
Urinary ketones	-ve

**Figure 1:**
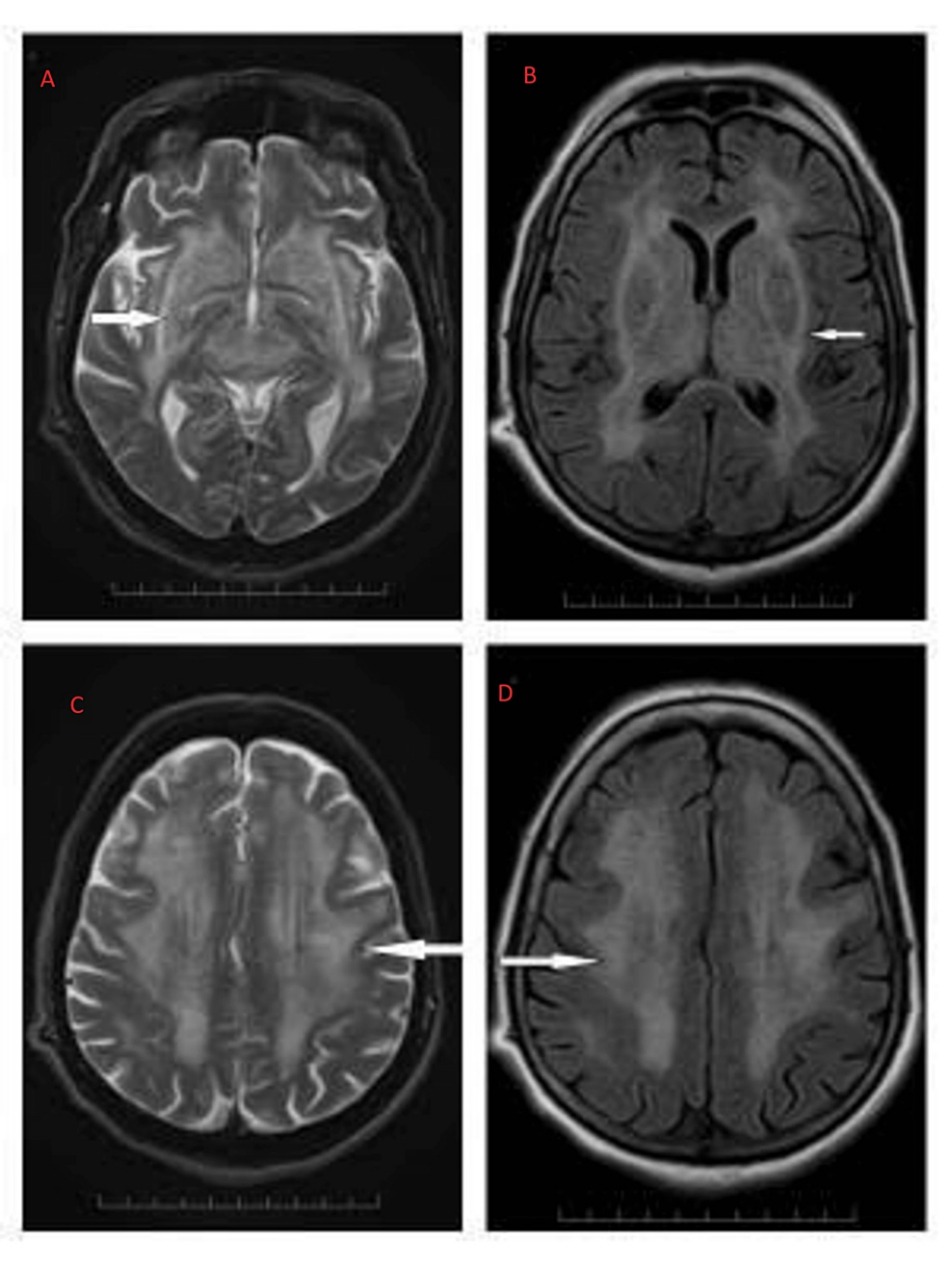
Brain MRI on admission: T2WI and FLAIR images in axial view showing bilateral symmetrical hyperintensities in BG and deep and subcortical white matter. Extensive confluent T2/FLAIR hyperintensities are noted. (A) The white matter surrounding BG is hyperintense on the T2WI sequence demarcating the lentiform nucleus from the surrounding structure. (B) FLAIR sequence also shows symmetrical hyperintensities involving BG. (C, D) T2/FLAIR images showing extensive deep and subcortical white matter involvement. MRI: magnetic resonance imaging; T2WI: T2-weighted imaging; FLAIR: fluid-attenuated inversion recovery; BG: basal ganglia.

**Figure 2:**
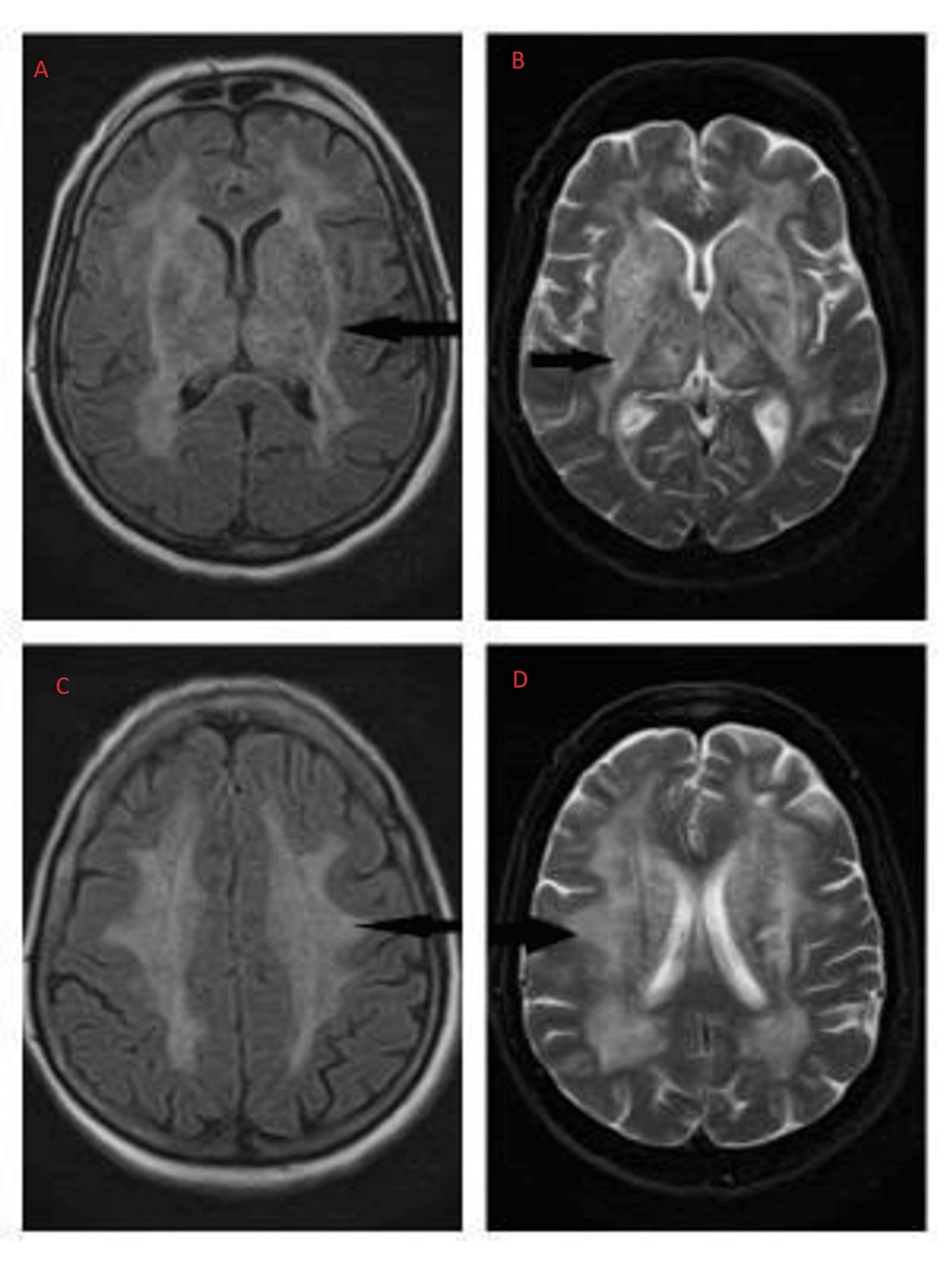
Brain MRI on follow-up: T2WI and FLAIR images in axial view showing minimal interval reduction in bilateral symmetrical hyperintensities of BG and deep and subcortical white matter. MRI performed on the fourth month of follow-up. (A, B) Symmetrical T2/FLAIR hyperintense signals in BG, thalami, and internal and external capsules. (C, D) Interval static extensive T2/FLAIR hyperintensities in bilateral periventricular, deep, and subcortical white matter. BG: basal ganglia.

MRI performed on admission showed a typical lentiform fork sign appearance (Figure 1). We started supportive measures with IV fluids and broad-spectrum antibiotics to treat the urinary tract infection (UTI). In light of the multi-disciplinary approach being used at our institution, we decided to manage the patient's renal impairment conservatively without starting hemodialysis. The patient responded well to IV hydration therapy. The patient's neurological symptoms improved gradually over two to three weeks. During a follow-up exam four months later, the patient's neurological status was back to normal with no acquired deficits; however, the subsequent MRI scan showed only slight improvement (Figure 2). Unfortunately, the metabolic status during the follow-up period is not known.

## Discussion

Metabolic leukoencephalitis, including UE, is common in patients presenting with altered mental status in the ER. However, these patients have a long list of differentials, making neuroimaging correlation crucial for narrowing the differential diagnosis, especially because adequate history and context are difficult to obtain in these patients. It is important to recognize the common imaging features of leukoencephalitis for an accurate and quick diagnosis.

We describe a case of classic MRI hyperintensities of bilateral BG in uremic encephalitis that shows dramatic clinical improvement with conservative management. The pathogenesis of these lesions is still not clear. The underlying combination of cytotoxic and vasogenic edema causing white matter patterns [5] is a commonly accepted mechanism and therefore explains the radiological regression of lesions upon clinical recovery. However, Sakurai et al. described a case with unilateral basal ganglion hyperintensity and lentiform fork sign because of uremia [1]. Their patient improved upon starting hemodialysis, and follow-up MRI showed resolution of edematous changes but persistent white matter lesions. CSF analysis also showed pleocytosis and elevated MBP in that patient. These findings may suggest an additional destructive and demyelinating pathology [1]. These findings are very similar to our case, but we do not have a cerebrospinal fluid (CSF) analysis to comment on neutrophilic pleocytosis. The persistence of metabolic acidosis can also be one of the possible reasons for the irreversibility of these lesions. Unfortunately, we do not have a metabolic profile from a follow-up time to comment on metabolic acidosis.

In the future, we suggest repeating the blood gas analysis for these patients. The long-term implications and prognosis of these various underlying pathological mechanisms are not clear and are an area where more studies need to be conducted. The hypothesis of metabolic acidosis is a common triggering factor behind these lesions, and their increased prevalence in Asian patients with diabetes also needs to be tested properly.

## Conclusions

Clinicians need to identify the lentiform fork sign on MRI, as it can signify the involvement of BG in UE. This sign can also be seen in any pathology leading to metabolic acidosis. Its incidence is reported to be higher in patients with diabetes. The presence of this sign, along with rapid clinical improvement following normalization of kidney function or dialysis initiation, confirms the diagnosis of UE in the appropriate clinical context. It should be noted that the absence of this sign does not mean that a diagnosis of uremic encephalitis is ruled out. These lesions seen on MRI scans typically resolve over time, but as in this case, their persistence has unknown implications for long-term prognosis.
